# Macrophage Immune-Redox Modulation by Phenylethanoid- and Flavonoid-Rich Botanical Extracts: NF-κB/iNOS–Nrf2 Crosstalk and the Proposed Role of *Buddleja globosa*

**DOI:** 10.3390/antiox15091158

**Published:** 2026-09-11

**Authors:** Yilka Mena-Linares, Humberto Vélez-Slimani, Luis A. Salazar

**Affiliations:** 1 Doctoral Program in Sciences, Major in Applied Cellular and Molecular Biology, Universidad de La Frontera, Temuco 4811230, Chile; y.mena03@ufromail.cl (Y.M.-L.); h.velez01@ufromail.cl (H.V.-S.); 2 Center of Molecular Biology and Pharmacogenetics, Department of Basic Sciences, Universidad de La Frontera, Temuco 4811230, Chile

**Keywords:** *Buddleja globosa*, macrophages, NF-κB, iNOS, Nrf2, oxidative stress, inflammation, verbascoside, flavonoids, botanical extracts

## Abstract

Macrophage activation depends on the balance between pro-inflammatory signaling and cytoprotective antioxidant responses. Persistent nuclear factor kappa B (NF-κB) and inducible nitric oxide synthase (iNOS) activity, together with insufficient nuclear factor erythroid 2-related factor 2 (Nrf2) signaling, promotes oxidative and nitrosative stress and contributes to chronic inflammation. Phenylethanoid- and flavonoid-rich botanical extracts may modulate this immune-redox network, but the available evidence is dispersed across plant species, extraction procedures, isolated compounds, and experimental models. This review examines the phytochemical composition and biological effects of these preparations, with particular emphasis on *Buddleja globosa* Hope. Evidence concerning verbascoside, luteolin, apigenin, quercetin derivatives, and other constituents is integrated with findings related to toll-like receptor 4/NF-κB signaling, iNOS-derived nitric oxide, inflammatory cytokines, reactive oxygen species, and Keap1–Nrf2-dependent cytoprotective responses. Direct evidence obtained with *B. globosa* extracts is distinguished from mechanistic findings generated using isolated constituents or related botanical preparations. Current studies support antioxidant and inflammation-modulating activities, but interpretation is limited by phytochemical variability, inconsistent dosing, incomplete cytotoxicity assessment, and limited use of macrophage-specific models. Future work should prioritize chemically standardized extracts, direct extract–constituent comparisons, pathway-specific validation, and formulation strategies that improve stability, reproducibility, and biological performance.

## 1. Introduction

Macrophages integrate microbial and tissue-derived signals to initiate, amplify, and resolve innate immune responses. Experimental exposure to bacterial lipopolysaccharide (LPS) induces a dynamic transcriptional program in macrophages that is largely coordinated by nuclear factor kappa B (NF-κB), rather than a uniform increase in inflammatory gene expression. The activation state, nuclear residence, and DNA-binding activity of the NF-κB p65 subunit influence the temporal sequence of cytokine production, including the early induction of tumor necrosis factor alpha (TNF-α) and the subsequent transition toward interleukin 10 (IL-10) expression. These findings illustrate that macrophage activation is determined not only by the magnitude of inflammatory signaling but also by its kinetics and regulatory feedback mechanisms [1,2].

One of the principal downstream outputs of NF-κB activation is inducible nitric oxide synthase (iNOS), which catalyzes the sustained production of nitric oxide (NO) during inflammatory stimulation. In macrophage models, LPS increases NF-κB activation, iNOS messenger RNA and protein expression, and NO production in a concentration- and time-dependent manner. Studies conducted in RAW 264.7 macrophages further demonstrated that LPS-induced NO production is associated with p65 nuclear translocation and is regulated through coordinated kinase signaling. The NF-κB/iNOS axis therefore represents a mechanistically connected component of macrophage inflammatory activation and a useful target for evaluating inflammation-modulating interventions [3,4].

Macrophage inflammatory signaling is closely coupled to the cellular redox state. Nuclear factor erythroid 2-related factor 2 (Nrf2) is negatively regulated under basal conditions by Kelch-like ECH-associated protein 1 (Keap1), which promotes its ubiquitination and proteasomal degradation. Oxidative or electrophilic stimulation disrupts this repression, allowing Nrf2 accumulation and activation of cytoprotective transcriptional programs. The functional importance of this pathway during innate immune activation has been demonstrated in Nrf2-deficient models, in which LPS exposure produced greater reactive oxygen species (ROS) accumulation and enhanced expression of inflammatory cytokines and chemokines. Conversely, pharmacological Nrf2 activation attenuated these responses in wild-type macrophages and protected mice against LPS-induced inflammatory injury [5,6].

The anti-inflammatory activity of Nrf2 is not limited to the indirect reduction in oxidative stress. Genetic activation of Nrf2 in macrophages was shown to interfere directly with the LPS-induced transcription of interleukin 6 (IL-6) and interleukin 1 beta (IL-1β) by limiting RNA polymerase II recruitment near these genes, independently of changes in intracellular ROS levels. Additional proteomic and functional analyses demonstrated that pharmacological or genetic Nrf2 activation remodels macrophage redox and intermediary metabolism, promotes mitochondrial adaptation, and suppresses components of the type I interferon response. These findings identify Nrf2 as an integrated regulator of antioxidant defense, inflammatory transcription, and macrophage immunometabolism, rather than exclusively as a conventional antioxidant pathway [7,8].

Within this mechanistic framework, phenylethanoid- and flavonoid-rich botanical preparations are of interest because their chemically diverse constituents may influence multiple nodes of macrophage signaling. *Buddleja globosa* Hope (*B. globosa*), commonly known in Chile as matico, is a South American medicinal species traditionally used for inflammatory and wound-related conditions and contains a complex mixture of phenylethanoid glycosides, flavonoids, iridoids, terpenoids, and sterols. Modern chromatographic profiling has identified verbascoside, forsythoside B, isoverbascoside, luteolin 7-O-glucoside, apigenin 7-O-glucoside, quercetin 3-O-glucoside, and related compounds, while bio-guided studies of serial leaf extracts have demonstrated antioxidant and anti-inflammatory activities in cellular and animal models [9,10].

Several major constituents of *B. globosa* provide mechanistic plausibility for its potential effects on macrophage immune-redox regulation. Verbascoside, also known as acteoside, inhibited LPS-induced iNOS expression in RAW 264.7 macrophages by suppressing activator protein 1 (AP-1) activation. In the same cellular model, luteolin and luteolin 7-O-glucoside reduced NO and prostaglandin E2 (PGE2) production, together with the expression of iNOS and cyclooxygenase-2 (COX-2), through differential regulation of NF-κB- and AP-1-associated signaling. Apigenin has likewise been shown to suppress pro-inflammatory cytokine expression in monocytes and macrophages by reducing NF-κB p65 phosphorylation [11,12,13].

However, findings obtained with purified constituents cannot be transferred uncritically to chemically complex botanical extracts. Glycosylation, extraction solvent, relative compound abundance, stability, cellular uptake, and interactions among constituents may substantially alter the resulting biological response. This distinction is supported by the greater inhibitory potency of luteolin compared with luteolin 7-O-glucoside in LPS-stimulated macrophages. It is also reinforced by experiments with an aqueous *B. globosa* leaf extract, which displayed antioxidant activity at low concentrations but became cytotoxic to fibroblasts at concentrations above 50 μg/mL. Therefore, suppression of inflammatory mediators should be interpreted only after confirming cellular viability and characterizing the preparation tested [13,14].

Accordingly, this review critically examines macrophage immune-redox modulation by phenylethanoid- and flavonoid-rich botanical extracts, with particular emphasis on *B. globosa*. Direct evidence obtained with complete extracts and fractions is distinguished from findings generated using isolated compounds or related botanical preparations. Particular attention is given to the NF-κB/iNOS–Nrf2 network, inflammatory cytokines, oxidative and nitrosative stress, extract standardization, concentration-dependent effects, and experimental factors that currently limit mechanistic and translational interpretation [9,10,11,12,13,14].

## 2. Literature Search and Evidence Classification

A targeted and iterative literature search was conducted using PubMed/MEDLINE, Scopus, and Web of Science Core Collection, covering the period from database inception to 20 July 2026. Searches were organized according to the thematic structure of the review and progressively refined using section-specific combinations of terms related to the botanical source, its principal constituents, macrophage biology, and immune-redox signaling. The search strategy combined terms related to the botanical source and its principal constituents, including “*Buddleja globosa*”, “matico”, “verbascoside”, “acteoside”, “phenylethanoid glycosides”, “luteolin”, “apigenin”, “quercetin”, “iridoids”, and “terpenoids”, with terms related to macrophage biology and immune-redox signaling, including “macrophage”, “monocyte”, “RAW 264.7”, “THP-1”, “lipopolysaccharide”, “TLR4”, “NF-κB”, “iNOS”, “NOS2”, “nitric oxide”, “Nrf2”, “NFE2L2”, “Keap1”, “HO-1”, “HMOX1”, “NQO1”, “reactive oxygen species”, “oxidative stress”, “cytokines”, and “inflammation”. Search strings were adapted to the indexing system of each database, and the reference lists of relevant primary studies and reviews were examined manually. Because the combinations of search terms were progressively refined according to the thematic focus of each section, no single fixed database-specific search string was applied across the entire review. The search strategy was developed to provide the transparency and methodological rigor expected for a narrative review without classifying the study as a formal systematic review [15,16].

Original research articles were considered eligible when they investigated: (i) complete *B. globosa* extracts or chemically defined fractions; (ii) purified compounds documented as constituents of *B. globosa*; or (iii) chemically characterized botanical preparations with direct relevance to macrophage inflammatory or redox signaling. Cellular, animal, human, and formulation studies were considered when they reported outcomes associated with NF-κB, iNOS and nitric oxide, Nrf2, reactive oxygen species, inflammatory cytokines, or inflammatory resolution. Review articles were used to contextualize the field and identify additional primary studies but were not treated as primary mechanistic evidence. Conference abstracts, editorials, studies lacking sufficient information about the preparation evaluated, and reports concerning other *Buddleja* species without a clear comparative rationale were excluded. No language restrictions were applied [17].

Because the literature search was conducted iteratively across the thematic sections of this narrative review, individual studies were frequently retrieved through multiple search combinations and could contribute to more than one section. Consequently, records were not prospectively tracked through a single screening process in which duplicate records were systematically removed, and retrospective reporting of the numbers identified, screened, and excluded would not accurately represent the search process.

To minimize mechanistic overinterpretation, we developed a four-category evidence classification specifically for the purposes of this review, based on the proximity of the retrieved evidence to *B. globosa* and to the central macrophage-focused research question. Category I comprised direct evidence obtained using complete *B. globosa* extracts or chemically defined fractions. Category II included constituent-based evidence derived from purified compounds confirmed in the plant, particularly verbascoside, luteolin derivatives, apigenin derivatives, and quercetin glycosides. Category III included comparative evidence from other chemically characterized botanical preparations containing related phenylethanoids or flavonoids. Category IV comprised translational evidence from animal models, pharmaceutical formulations, hydrogels, scaffolds, and other delivery systems. These categories were used to indicate the origin and degree of proximity of each mechanistic inference rather than to establish a formal clinical hierarchy of evidence.

For each study, information was organized according to botanical identity, plant part, extraction or fractionation method, phytochemical characterization, experimental model, inflammatory stimulus, concentration or dose, exposure time, cellular viability, molecular endpoints, and pathway-specific validation. Findings were synthesized narratively according to intervention type and mechanistic target. Results obtained from cell-free antioxidant assays were not interpreted as evidence of intracellular Nrf2 activation, and reductions in inflammatory mediators were not considered pathway-specific unless supported by appropriate molecular measurements. Similarly, effects demonstrated using isolated compounds were not attributed directly to complete *B. globosa* extracts unless comparative experimental evidence was available [18]. ChatGPT (OpenAI, San Francisco, CA, USA, GPT-5, accessed on 10 July 2026) was used for literature organization, manuscript restructuring, drafting support, and language refinement.

## 3. Macrophage Immune-Redox Signaling

Macrophage activation involves the coordinated reprogramming of receptor-mediated signaling, transcription, metabolism, and cellular redox balance. These processes are highly interconnected: inflammatory stimuli modify the macrophage enhancer landscape and activate stimulus-specific transcriptional programs, while metabolic intermediates and ROS function as intracellular signals that influence cytokine production and cellular adaptation. Consequently, immune and redox responses should not be interpreted as independent processes, but as components of an integrated network that determines the intensity, duration, and eventual resolution of macrophage activation [19,20].

### 3.1. Macrophage Activation and Inflammatory Phenotypes

Macrophages are functionally heterogeneous cells involved in pathogen recognition, phagocytosis, cytokine production, antigen presentation, tissue remodeling, and restoration of homeostasis. Their functional state is shaped by their developmental origin and by the combination of microbial products, cytokines, metabolites, extracellular matrix signals, and damaged-cell components present in the surrounding microenvironment. Transcriptional profiling of human monocyte-derived macrophages has shown that differentiation and subsequent activation generate distinct gene-expression programs associated with inflammatory defense, immune regulation, lipid metabolism, and tissue repair [21].

Macrophage activation has traditionally been described using the M1/M2 classification. In this model, exposure to interferon gamma (IFN-γ), either alone or combined with LPS, generates an M1-like state characterized by the production of inflammatory mediators, antimicrobial activity, and increased expression of iNOS. In contrast, stimulation with interleukin 4 (IL-4) or interleukin 13 (IL-13) promotes an M2-like program associated with immune regulation, extracellular matrix remodeling, and tissue repair. Although this classification remains useful for controlled experimental comparisons, the resulting states represent simplified responses to specific stimuli rather than fixed macrophage populations [21].

Macrophage phenotypes can change progressively and reversibly when the cytokine environment is modified. Sequential exposure to inflammatory and regulatory stimuli produces combinations of functional properties that cannot be assigned exclusively to a single M1 or M2 category. This plasticity allows macrophages to initially participate in microbial elimination and inflammatory amplification and subsequently acquire functions related to clearance of damaged cells, suppression of inflammation, and tissue restoration [22].

A broader transcriptomic analysis of human macrophages exposed to numerous activation signals demonstrated a continuous spectrum of stimulus-dependent states rather than two opposing phenotypes. Different combinations of cytokines, fatty acids, microbial ligands, and inflammatory mediators generated partially overlapping transcriptional modules controlled by distinct regulatory networks. These findings indicate that macrophage activation is multidimensional and that the simultaneous expression of conventionally classified M1- and M2-associated markers does not necessarily represent an experimental inconsistency [23].

The phenotype adopted by macrophages also depends on tissue-specific signals that influence chromatin accessibility and enhancer selection. Comparative analysis of resident macrophages demonstrated that local microenvironments establish distinct transcriptional and epigenetic identities, and that these programs can be partially reconfigured when cells are transferred to a different tissue environment. Therefore, responses observed in RAW 264.7 cells, THP-1-derived macrophages, primary bone marrow-derived macrophages, or tissue-resident populations should not be considered directly interchangeable [24].

Inflammatory activation is additionally accompanied by metabolic remodeling that contributes directly to macrophage function. LPS-stimulated macrophages redirect mitochondrial activity toward succinate oxidation and ROS production, reinforcing the expression of inflammatory mediators. Pharmacological restriction of this metabolic pathway promotes a less inflammatory response, demonstrating that cellular metabolism is not merely a consequence of activation but an active regulator of macrophage phenotype. Accordingly, the effects of botanical extracts should be evaluated using defined stimuli, exposure times, viability measurements, molecular endpoints, and functional responses rather than inferred from changes in a single polarization marker [25].

### 3.2. TLR4, IκB, and NF-κB Signaling

TLR4 is the principal receptor responsible for the recognition of LPS from Gram-negative bacteria. The central role of TLR4 was established through the identification of mutations in the *Tlr4* gene that rendered mouse strains poorly responsive to LPS. At the molecular level, LPS binds to myeloid differentiation factor 2 (MD-2), which associates with TLR4 and promotes the formation of an activated receptor dimer. This receptor complex converts extracellular recognition of bacterial endotoxin into intracellular inflammatory signaling [26,27].

At the plasma membrane, activated TLR4 recruits myeloid differentiation primary response 88 (MyD88), initiating a kinase cascade that converges on the IκB kinase (IKK) complex. The importance of this adaptor is demonstrated by the markedly impaired response of MyD88-deficient mice and immune cells to LPS. Activated IKK phosphorylates inhibitor of NF-κB alpha (IκB-α), promoting its ubiquitination and proteasomal degradation. This process releases NF-κB dimers retained in the cytoplasm and allows the p65-containing complex to translocate into the nucleus, where it regulates an inflammatory transcriptional program that includes TNF-α, IL-6, IL-1β, iNOS, and COX-2 [28,29].

TLR4 also activates a MyD88-independent signaling branch mediated by TIR-domain-containing adapter-inducing interferon-β (TRIF). Genetic disruption of TRIF impairs TLR4-dependent activation of interferon regulatory factor 3 (IRF3) and the production of interferon-β, while also modifying the delayed phase of inflammatory gene expression. The MyD88 and TRIF pathways should therefore not be viewed as completely isolated cascades, because both contribute to the timing and composition of the macrophage response to LPS [30].

The relative contribution of these adaptor pathways differs according to the endpoint examined. Live-cell analysis of LPS-stimulated macrophages showed that MyD88 is the principal driver of rapid NF-κB nuclear translocation. However, efficient activation of the TNF-α promoter requires signaling contributions from both MyD88 and TRIF. Thus, the observation of reduced NF-κB nuclear localization, cytokine expression, or promoter activity may reflect effects at different levels of the TLR4 signaling network and should not automatically be interpreted as inhibition of the same molecular target [31].

NF-κB activation is also regulated by mechanisms that limit the magnitude and duration of the inflammatory response. Experimental disruption of IKKα in macrophages resulted in prolonged NF-κB activity and delayed inflammatory resolution, demonstrating that components of the pathway can exert both activating and regulatory functions. For this reason, NF-κB should not be interpreted as a simple binary switch. The timing of IκB-α degradation, p65 nuclear accumulation, DNA binding, transcriptional activity, and subsequent negative feedback must be considered when evaluating the effects of phytochemicals or botanical extracts on this pathway [32].

### 3.3. iNOS, Nitric Oxide, and Nitrosative Stress

iNOS is a high-output enzyme induced primarily at the transcriptional level following macrophage exposure to microbial products and inflammatory cytokines. Once expressed, it catalyzes sustained NO production from L-arginine and molecular oxygen. Unlike constitutively expressed nitric oxide synthases, iNOS can remain active for prolonged periods and generate concentrations of NO sufficient to modify microbial, cellular, and extracellular targets. Its original cloning from activated murine macrophages established iNOS as a central component of the immunologically induced effector response [33].

The biological consequences of iNOS activation depend on the intensity, location, and duration of NO production. Genetic deletion of iNOS impaired the ability of mice to restrict *Listeria monocytogenes* replication and reduced macrophage-mediated control of tumor cells, confirming its contribution to antimicrobial and cytotoxic defense. However, the same experiments showed that iNOS-derived NO also participates in systemic responses to endotoxin. These findings demonstrate that iNOS is neither exclusively protective nor exclusively damaging, and that its function is determined by the inflammatory context in which it is activated [34].

NO can react rapidly with superoxide to form peroxynitrite (ONOO^−^), a highly reactive member of the reactive nitrogen species (RNS) family. In activated macrophages, limited L-arginine availability can promote the generation of both superoxide and NO by iNOS, favoring ONOO^−^ formation. Sustained nitrosative stress may subsequently modify protein thiols and tyrosine residues, alter mitochondrial activity, promote lipid oxidation, and damage nucleic acids. Consequently, a prolonged increase in NO production may amplify cellular injury even when iNOS is initially induced as part of a protective antimicrobial response [35].

The connection between NF-κB activation and iNOS expression is supported by the presence of multiple functional κB elements in the regulatory region of the *NOS2* gene. Cytokine-induced iNOS transcription and NO production were markedly reduced when NF-κB activation or its promoter-binding sites were disrupted. Nevertheless, iNOS expression is also influenced by additional transcription factors and by cell-specific regulatory elements. A reduction in iNOS should therefore not automatically be interpreted as evidence of direct NF-κB inhibition unless changes in IκB-α, p65 nuclear translocation, DNA binding, or NF-κB-dependent transcription are demonstrated experimentally [36].

The assessment of iNOS activity in botanical studies requires similar caution. Measurement of nitrite accumulation in culture medium provides an indirect estimate of NO production but does not identify the molecular level at which an intervention acts. Reduced nitrite may result from lower *NOS2* transcription, decreased iNOS protein abundance, direct interference with enzymatic activity, chemical scavenging of NO, altered substrate availability, or loss of viable cells. Mechanistic evaluation should therefore combine cellular viability with measurements of *NOS2* expression, iNOS protein, NO production, and upstream signaling under clearly defined concentrations and exposure times [33,35,36].

### 3.4. Keap1–Nrf2–HO-1/NQO1 Signaling

Under basal conditions, Keap1 represses Nrf2 activity and maintains low intracellular Nrf2 abundance. Electrophilic or oxidative modification of Keap1 disrupts this repression, permitting Nrf2 accumulation and nuclear translocation. Nuclear Nrf2 associates with small Maf proteins and binds to the antioxidant response element (ARE) within regulatory regions of cytoprotective genes. This system allows cells to detect changes in their chemical and redox environment and rapidly modify gene expression without requiring a proportional increase in *NFE2L2* transcription [37].

The importance of this pathway in macrophages was established using cells obtained from Nrf2-deficient mice. In the absence of Nrf2, macrophages failed to induce a coordinated group of oxidative stress-responsive genes and showed reduced expression of proteins involved in electrophile detoxification, redox control, and cystine transport. These findings positioned Nrf2 as a regulator of a broad cytoprotective program rather than a transcription factor controlling a single antioxidant enzyme [38].

HO-1 and NQO1 are frequently used as downstream indicators of Nrf2 activity. HO-1 catalyzes the degradation of heme and generates products capable of influencing oxidative balance and inflammatory signaling. NQO1 catalyzes the reduction in quinones and limits their participation in redox cycling. Their induction can therefore improve cellular resistance to oxidative and electrophilic injury. However, changes in HO-1 or NQO1 alone do not establish activation of the complete pathway because their expression may also be influenced by Nrf2-independent transcriptional mechanisms. Appropriate pathway assessment should include Nrf2 stabilization or nuclear localization together with several ARE-regulated targets [37,38].

Macrophage metabolism provides an additional mechanism for activating this pathway. Itaconate, an endogenous metabolite that accumulates after LPS stimulation, can alkylate specific Keap1 cysteine residues, allowing Nrf2-dependent induction of HO-1, NQO1, and other cytoprotective genes. In mouse and human macrophages, this response was associated with reduced inflammatory cytokine production and protection against LPS-induced lethality. These observations connect metabolic remodeling directly with electrophile sensing and Nrf2-mediated inflammatory control [39].

For botanical extracts, an increase in total Nrf2 protein cannot by itself demonstrate functional activation. A complete evaluation should determine whether Nrf2 accumulates in the nucleus, binds or activates ARE-dependent regulatory regions, and increases downstream gene or protein expression. Causal evidence may additionally require Nrf2-deficient cells, gene silencing, pathway inhibitors, or comparison with a validated Nrf2 activator. These controls are particularly important for polyphenol-rich extracts because their constituents may alter ROS measurements directly or interfere chemically with colorimetric and fluorometric assays [37,38,39].

### 3.5. Functional Crosstalk Between NF-κB, iNOS, and Nrf2

The NF-κB/iNOS and Nrf2 pathways form an interconnected regulatory network rather than two independent signaling systems. Increased ROS can facilitate inflammatory signaling, whereas Nrf2-dependent cytoprotective responses can restrict the redox conditions that sustain NF-κB activation. In experimental sepsis, Nrf2 deficiency produced exaggerated NF-κB and IRF3 activation, increased expression of inflammatory genes, greater tissue injury, and reduced survival. These results demonstrate that basal and inducible Nrf2 activity contributes to maintaining an appropriately controlled innate immune response [40].

Experiments using primary macrophages from wild-type and Nrf2-deficient mice have also shown that the anti-inflammatory effects of bioactive compounds may depend on an intact Nrf2 pathway. Sulforaphane reduced LPS-induced iNOS, COX-2, and inflammatory mediator production more effectively in wild-type macrophages than in Nrf2-deficient cells [41]. Similarly, the ability of docosahexaenoic and eicosapentaenoic acids to suppress iNOS, COX-2, TNF-α, IL-1β, and IL-6 was associated with Nrf2 activation and was attenuated when Nrf2 was absent [42]. These studies illustrate the value of genetic models for distinguishing Nrf2-dependent effects from general antioxidant or anti-inflammatory activity.

HO-1 provides a mechanistic link between Nrf2 activation and the regulation of the NF-κB/iNOS axis. In macrophages, LPS initially induced iNOS and was followed by increased HO-1 expression. Pharmacological induction of HO-1 suppressed subsequent iNOS expression, whereas this negative feedback response was weakened in Nrf2-deficient macrophages. The temporal sequence indicates that the Nrf2/HO-1 response may function as an endogenous mechanism that limits sustained NO production after the initial inflammatory phase [43].

**Figure 1:**
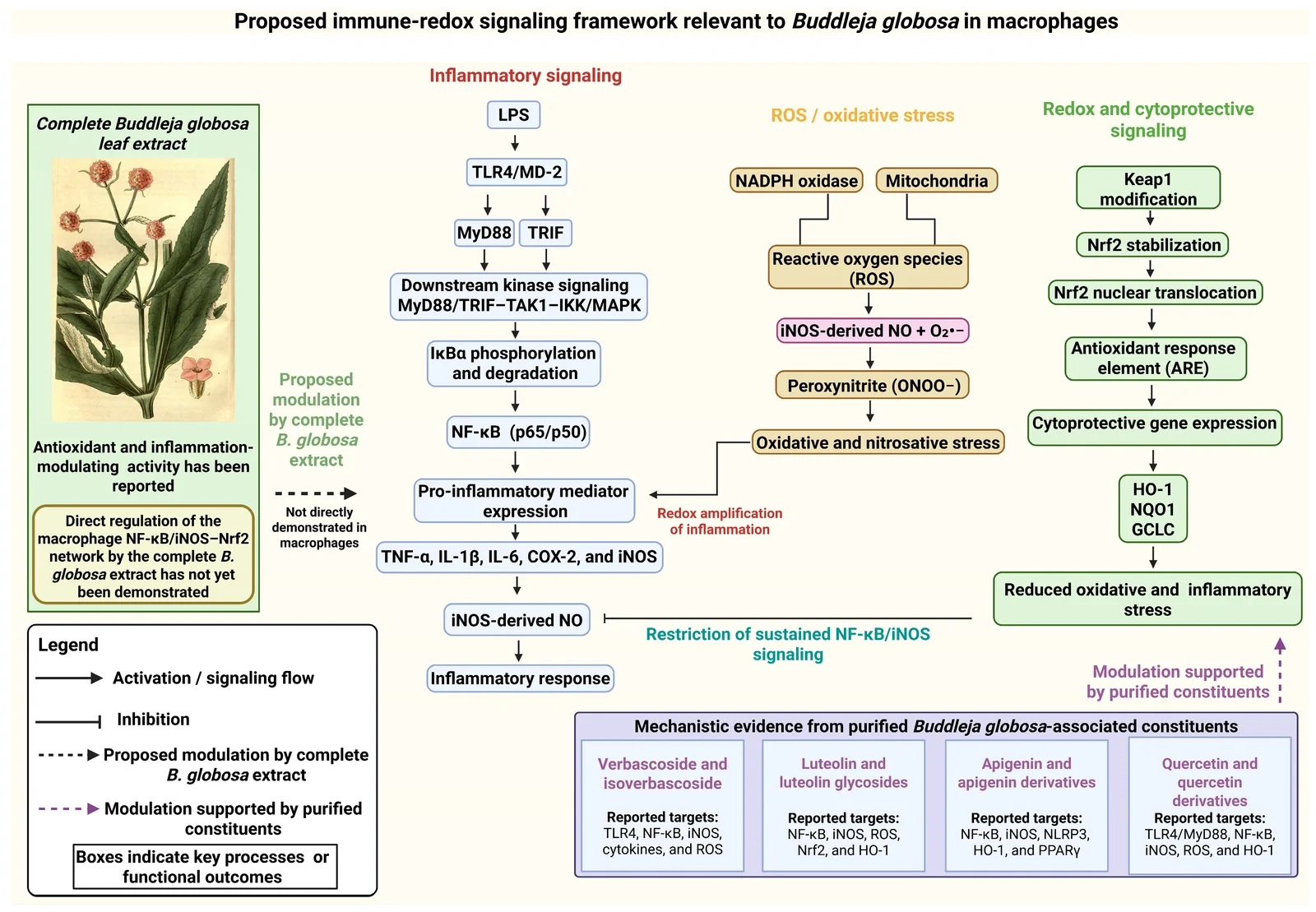
Proposed macrophage immune-redox framework linking TLR4/NF-κB/iNOS signaling with Keap1–Nrf2-mediated cytoprotective responses and the potential modulation exerted by *Buddleja globosa*-associated constituents. Lipopolysaccharide recognition by the TLR4/MD-2 complex activates MyD88- and TRIF-dependent signaling, leading to IKK activation, IκB-α degradation, NF-κB nuclear translocation, and the expression of inflammatory cytokines, COX-2, and *NOS2*/iNOS. Increased reactive oxygen species and iNOS-derived nitric oxide contribute to oxidative and nitrosative stress, including peroxynitrite formation. Oxidative or electrophilic modification of Keap1 stabilizes Nrf2 and promotes its nuclear translocation, resulting in antioxidant response element-dependent expression of HO-1, NQO1, and other cytoprotective genes that may limit sustained NF-κB/iNOS signaling. Verbascoside-related phenylethanoids and luteolin, apigenin, and quercetin derivatives have been reported to influence selected nodes of this network in macrophage or related experimental models. Solid arrows represent activation or signaling flow, blunt-ended lines indicate inhibition, black dashed arrows indicate proposed extract-level modulation, and purple dashed arrows indicate constituent-supported modulation. Regulation of this network by complete *B. globosa* extracts remains proposed but has not yet been directly demonstrated in macrophages. Created in BioRender. Vélez Slimani, H. (2026). https://BioRender.com/pha3m8c (accessed on 8 September 2026). Abbreviations: ARE, antioxidant response element; COX-2, cyclooxygenase-2; GCLC, glutamate-cysteine ligase catalytic subunit; HO-1, heme oxygenase-1; IKK, inhibitor of nuclear factor kappa B kinase; IκB-α, inhibitor of nuclear factor kappa B alpha; IL-1β, interleukin 1 beta; IL-6, interleukin 6; iNOS, inducible nitric oxide synthase; Keap1, Kelch-like ECH-associated protein 1; LPS, lipopolysaccharide; MAPK, mitogen-activated protein kinase; MD-2, myeloid differentiation factor 2; MyD88, myeloid differentiation primary response 88; NADPH, nicotinamide adenine dinucleotide phosphate; NF-κB, nuclear factor kappa B; NO, nitric oxide; NOS2, nitric oxide synthase 2; NQO1, NAD(P)H quinone oxidoreductase 1; Nrf2, nuclear factor erythroid 2-related factor 2; O_2_•^−^, superoxide radical; ONOO^−^, peroxynitrite; p50, NF-κB1 subunit; p65, RelA subunit of NF-κB; ROS, reactive oxygen species; TAK1, transforming growth factor beta-activated kinase 1; TLR4, toll-like receptor 4; TNF-α, tumor necrosis factor alpha; TRIF, TIR-domain-containing adapter-inducing interferon-β.

The NF-κB/iNOS and Nrf2 pathways should not be interpreted as a simple reciprocal switch. During LPS stimulation, pro-inflammatory signaling and compensatory Nrf2-dependent responses may occur simultaneously, and the resulting macrophage phenotype depends on the timing and magnitude of each pathway. Therefore, reductions in ROS, NO, and inflammatory cytokines accompanied by increased Nrf2, HO-1, or NQO1 expression are consistent with immune-redox modulation, but do not independently demonstrate direct pathway crosstalk. Establishing this interaction requires temporal analyses and pathway-specific interventions, such as Nrf2 inhibition or genetic deficiency, to determine whether suppression of NF-κB or iNOS depends on Nrf2 activation. This distinction is essential when assessing whether *B. globosa* extracts directly regulate the NF-κB/iNOS–Nrf2 network or whether the proposed mechanisms are inferred from isolated constituents or related botanical preparations [39,40,43]. The functional relationships between TLR4-dependent NF-κB/iNOS activation, Keap1–Nrf2-mediated cytoprotection, and the mechanistic evidence attributed to *B. globosa*-associated constituents are summarized in Figure 1.

## 4. Phytochemical Basis and Standardization of *Buddleja globosa*

The interpretation of the biological effects attributed to *Buddleja globosa* requires consideration of the chemical composition and standardization of the tested preparation. This section examines the main sources of phytochemical variability and the analytical criteria needed to relate extract composition to macrophage immune-redox responses.

### 4.1. Botanical and Pre-Analytical Sources of Variability

*Buddleja globosa* leaf preparations are not chemically uniform. Differences in provenance, cultivation status, soil moisture, growing season, harvest frequency, and postharvest handling can alter biomass production and the accumulation of flavonoids, tannins, and other bioactive constituents. Importantly, greater leaf productivity does not necessarily correspond to higher phytochemical content. Species authentication alone is therefore insufficient to define the chemical and pharmacological identity of the botanical material [44,45].

Drying, storage, particle size, solvent composition, plant-to-solvent ratio, extraction temperature, extraction time, and fractionation procedure further determine metabolite recovery. Consequently, aqueous, hydroalcoholic, methanolic, and nonpolar preparations should not be treated as chemically or pharmacologically equivalent. Studies intended to relate *Buddleja globosa* preparations to macrophage immune-redox signaling should report botanical authentication, plant organ and provenance, collection and postharvest conditions, extraction parameters, extraction yield, storage conditions, and quantitative phytochemical characterization [9].

### 4.2. Major Phenylethanoid and Flavonoid Markers

Verbascoside, also known as acteoside, is one of the principal phenylethanoid glycosides documented in *Buddleja globosa* leaves and represents a useful quantitative marker for polar leaf extracts. However, its abundance varies according to the harvesting season and extraction procedure, and changes in verbascoside content have been associated with differences in the biological activity of the resulting preparations [46,47]. Flavonoids and related phenolic compounds also contribute to the chemical identity of these extracts. Chromatographic analysis of a methanolic leaf preparation identified luteolin, quercetin, rutin, caffeic acid, and other phenolic constituents, while demonstrating that fractionation substantially modifies their relative abundance [48].

Neither verbascoside concentration nor total phenolic content alone adequately represents the chemical complexity of a complete extract. Verbascoside stability depends on pH, storage, and formulation conditions, while flavonoid glycosylation can modify polarity, hydrolysis, cellular uptake, and biological availability [49,50,51]. Mechanistic studies should therefore combine the quantification of representative phenylethanoid and flavonoid markers with a broader chromatographic fingerprint. Findings obtained with luteolin, apigenin, quercetin, or other purified aglycones should not be attributed directly to their glycosylated derivatives or to complete *Buddleja globosa* extracts without comparative experimental evidence.

### 4.3. Extraction-Dependent Composition and Analytical Standardization

Extraction solvent and fractionation determine the phytochemical composition of the resulting *Buddleja globosa* preparation. Sequential extraction of leaves with solvents of different polarity generated chemically and biologically distinct fractions. Polar methanolic preparations were enriched in phenylethanoid glycosides and flavonoids, whereas hexane- and dichloromethane-derived fractions contained greater proportions of triterpenoids and sterols. Consequently, aqueous, hydroalcoholic, methanolic, and nonpolar extracts should not be compared as equivalent interventions, even when obtained from the same species and plant organ [10].

Post-extraction processing can further modify extract concentration, solubility, constituent stability, and biological performance. Spray drying of a standardized hydroalcoholic *Buddleja globosa* extract, with or without solubility-enhancing excipients, altered its water dispersibility and antimicrobial activity despite the use of the same starting preparation [52]. Standardization for macrophage studies should therefore extend beyond total phenolic content or a single marker compound and include extraction yield, quantitative analysis of representative constituents, chromatographic fingerprinting, storage stability, residual solvent or excipient information, and the concentration of extract actually delivered to the cells. The preparation-dependent chemical characteristics and standardization requirements most relevant to macrophage immune-redox studies are summarized in Table 1.

## 5. Direct Evidence from *Buddleja globosa* Extracts and Fractions

Direct evidence concerning *Buddleja globosa* has been generated using chemically diverse extracts, fractions, and experimental models. This section evaluates these findings according to the preparation tested, the biological relevance of the assay, and the extent to which the reported effects support macrophage-specific immune-redox mechanisms.

### 5.1. Antioxidant Activity and Redox-Related Effects

Polar *Buddleja globosa* leaf extracts have demonstrated antioxidant activity in chemical and biomolecular systems. Sequential extraction studies showed that methanolic preparations containing verbascoside, luteolin 7-O-glucoside, and apigenin 7-O-glucoside scavenged 2,2-diphenyl-1-picrylhydrazyl radicals and limited superoxide generation and lipid peroxidation [10]. Additional experiments using microsomal systems demonstrated protection against lipid peroxidation, protein thiol oxidation, and oxygen consumption induced by oxygen-derived radicals. However, responses obtained with the synthetic DPPH radical did not fully reproduce protection of biological macromolecules, indicating that chemical radical-scavenging assays and activity in biologically organized systems should not be interpreted as equivalent outcomes [55].

Antioxidant performance varies according to the botanical material and preparation tested. Leaf provenance, harvesting period, irrigation, and drying conditions influenced polyphenol content and the protection of microsomal lipids and thiol groups [56]. Infusions and aqueous leaf extracts also showed antioxidant activity, but the available evidence was generated predominantly using chemical assays, epithelial or placental cell lines, and human erythrocytes rather than macrophages [54,57]. In erythrocytes, low extract concentrations reduced hypochlorous acid-induced hemolysis, while direct membrane interactions and morphological alterations were also observed under selected conditions [54]. These findings indicate that antioxidant protection may coexist with concentration-dependent cellular effects.

The current evidence therefore supports radical-scavenging activity and protection against oxidative damage to lipids, proteins, and cellular membranes. Nevertheless, these findings do not demonstrate Nrf2 activation or the regulation of macrophage-derived ROS. Establishing a macrophage immune-redox mechanism will require chemically standardized extracts, viability testing under matched exposure conditions, intracellular ROS measurements, Nrf2 nuclear localization, and analysis of HO-1, NQO1, and additional antioxidant response element-regulated targets.

### 5.2. Modulation of Inflammatory Mediators

Direct anti-inflammatory evidence for *Buddleja globosa* has been generated using chemically distinct preparations and predominantly non-macrophage experimental systems. Lipophilic root extracts inhibited cyclooxygenase and 5-lipoxygenase activity in stimulated rat peritoneal leukocytes, and bioassay-guided fractionation identified buddledin A among the constituents capable of suppressing eicosanoid generation [58]. Sequential leaf extracts also attenuated acute inflammation in vivo. Methanolic preparations reduced carrageenan-induced paw edema after oral administration and 12-*O*-tetradecanoylphorbol-13-acetate-induced ear edema after topical application, while hexane- and dichloromethane-derived fractions displayed activity associated with mixtures containing amyrins, phytosterols, and β-sitosterol glycoside [10]. Seasonal differences in verbascoside abundance were additionally associated with changes in the antinociceptive activity of alcoholic leaf extracts [47].

These studies establish pharmacological activity against eicosanoid-generating enzymes and acute inflammatory responses, but they do not demonstrate direct regulation of macrophage cytokines or the NF-κB/iNOS–Nrf2 network. Evidence obtained from nociception, edema, or isolated leukocyte enzyme assays should therefore be interpreted as support for anti-inflammatory plausibility rather than as proof of pathway-specific immune-redox modulation.

### 5.3. Evidence in Cellular Models and the Lack of Direct Macrophage Studies

Direct cellular evidence for *Buddleja globosa* has been generated predominantly in non-immune models. An aqueous leaf extract and chemically separated fractions protected human dermal fibroblasts against hydrogen peroxide-induced oxidative injury, while fractionation identified several flavonoids and caffeic acid derivatives contributing to the response [14]. A polyphenol-rich ethanolic leaf extract also inhibited human platelet aggregation induced by collagen, convulxin, and adenosine diphosphate. This effect was associated with reduced phosphorylation of phospholipase C gamma 2 and protein kinase C, demonstrating that a complete *Buddleja globosa* extract can modify intracellular signaling in human cells [59]. Nevertheless, fibroblast protection and platelet signaling cannot be extrapolated directly to macrophage inflammatory pathways.

Within the search strategy described in Section 2, no eligible primary study identified up to 20 July 2026 directly evaluated a complete, chemically standardized *Buddleja globosa* extract in macrophages with pathway-specific assessment of the NF-κB/iNOS–Nrf2 network. Evidence is therefore unavailable for extract-mediated changes in NF-κB nuclear translocation, *NOS2* expression, iNOS protein abundance, nitric oxide production, Nrf2 activation, or macrophage cytokine release. Regulation of the macrophage NF-κB/iNOS–Nrf2 network by the complete extract remains a biologically plausible hypothesis rather than an established mechanism.

### 5.4. Concentration-Dependent Effects and Cytotoxicity

The biological compatibility of *Buddleja globosa* preparations depends on extract composition, concentration, exposure time, vehicle, and cellular model. A methanolic leaf extract and an iridoid-enriched fraction showed trypanocidal IC_50_ values of 280 and 358 μg/mL, respectively, while their IC_50_ values in mammalian Vero cells exceeded 5000 μg/mL [48]. Although these findings indicate a favorable selectivity interval in that experimental system, Vero-cell compatibility does not establish safety in activated macrophages. Similarly, aqueous leaf extracts protected human erythrocytes against hypochlorous acid-induced damage at low concentrations but also produced membrane interactions and morphological alterations under selected conditions [54]. Formulation studies further demonstrated that ethanol, spray drying, and solubility-enhancing excipients can modify extract dispersibility and biological activity, emphasizing the need for matched vehicle and excipient controls [52].

Macrophage studies should establish a non-cytotoxic concentration range under the same stimulation, exposure time, and culture conditions used for molecular analyses. Reductions in nitric oxide, reactive oxygen species, or cytokine release should not be interpreted as anti-inflammatory effects when accompanied by decreased cell number, metabolic impairment, apoptosis, or membrane damage. Viability and membrane-integrity measurements should therefore precede pathway-specific evaluation and be reported together with the concentrations used to assess NF-κB, iNOS, Nrf2, and their downstream targets.

### 5.5. Strength and Limitations of the Direct Evidence

Direct studies support antioxidant, anti-inflammatory, membrane-protective, and cell-signaling effects of chemically diverse *Buddleja globosa* preparations. However, the evidence is distributed across different plant organs, extraction procedures, concentrations, formulations, and predominantly non-macrophage experimental systems. Chemical antioxidant assays, acute edema models, fibroblasts, erythrocytes, platelets, and microorganisms establish pharmacological plausibility but do not demonstrate direct regulation of macrophage NF-κB, iNOS, or Nrf2 signaling.

The central unresolved question is whether a chemically standardized *Buddleja globosa* extract can modulate the NF-κB/iNOS–Nrf2 network in macrophages at non-cytotoxic concentrations.

## 6. Major Bioactive Constituents and Macrophage Signaling

The limited pathway-specific evidence available for complete *B. globosa* extracts makes it necessary to examine their major constituents separately. Studies using purified molecules can identify plausible molecular targets and clarify whether individual phytochemicals influence macrophage inflammatory or antioxidant signaling. However, constituent-level activity cannot be assumed to reproduce the biological response of the complete extract. The concentration of an isolated compound used experimentally may differ substantially from its actual abundance within the botanical preparation, while glycosylation, stability, solubility, cellular uptake, metabolism, and interactions with other constituents may modify its biological activity. Experimental extract–constituent comparisons further demonstrate that complete botanical preparations, enriched fractions, and isolated compounds can produce quantitatively or even directionally different macrophage responses [60,61]. Constituent-based findings are therefore interpreted here as mechanistic support rather than direct evidence of pathway regulation by the complete *B. globosa* extract.

### 6.1. Verbascoside and Related Phenylethanoids

Verbascoside has demonstrated inflammation- and redox-modulating activity in human monocytic cells. In THP-1 cells, treatment with 100 μM verbascoside reduced iNOS expression and activity, and extracellular superoxide production, and altered the activities of superoxide dismutase, catalase, and glutathione peroxidase [62]. These findings indicate regulation of nitrosative and oxidative responses, although Nrf2 nuclear translocation and downstream target expression were not evaluated.

Acteoside has also influenced macrophage-related responses under different inflammatory stimuli. In primary bone marrow macrophages and RAW 264.7 cells exposed to the receptor activator of NF-κB ligand, acteoside reduced intracellular ROS, inhibited NF-κB and mitogen-activated protein kinase signaling, and suppressed osteoclast differentiation through reduced c-Fos and nuclear factor of activated T cells 1 expression [63]. In THP-1 cells, acteoside also attenuated interleukin-32-induced caspase-1 and NF-κB activation. Following macrophage-like differentiation and LPS stimulation, treatment reduced nitric oxide and the production of TNF-α, IL-1β, IL-6, and IL-8 [64]. These studies support activity across osteoclastogenic, inflammasome-associated, and endotoxin-induced responses, but the experimental contexts are not directly interchangeable.

Isoacteoside, also known as isoverbascoside, provides more direct evidence of an upstream macrophage target. In mouse macrophages, the compound interfered with LPS-induced TLR4 dimerization and inhibited the MyD88–TAK1–NF-κB/mitogen-activated protein kinase and TRIF signaling branches. These changes were accompanied by reduced expression of iNOS, COX-2, TNF-α, IL-6, and IL-1β [65]. The finding is relevant because isoverbascoside has been identified in *Buddleja globosa*.

Evidence linking verbascoside to Nrf2 has been obtained outside macrophage models. In hydrogen peroxide-exposed C2C12 myoblasts and myotubes, verbascoside reduced intracellular ROS, promoted Nrf2 phosphorylation and nuclear translocation, increased HO-1 transcription, and improved mitochondrial spare respiratory capacity [66]. Cytotoxicity was observed in myoblasts at concentrations of 250 μM or higher, emphasizing the influence of concentration and cellular state. The available evidence therefore supports suppression of TLR4/NF-κB signaling, iNOS, inflammatory cytokines, and ROS, together with plausible Nrf2/HO-1 activation.

### 6.2. Luteolin and Luteolin Glycosides

Luteolin directly regulates inflammatory and redox-sensitive signaling in macrophage models. In LPS-stimulated MH-S alveolar macrophages and RAW 264.7 cells, luteolin reduced NO, PGE2, TNF-α, and IL-6 production; decreased iNOS and COX-2 expression; and inhibited NF-κB and AP-1 DNA-binding activity. Reduced intracellular ROS generation further indicated that its effects involved both inflammatory transcription and cellular redox regulation [67].

Evidence relevant to the glycosylated constituent identified in *Buddleja globosa* was obtained by comparing luteolin with luteolin 7-O-glucoside in RAW 264.7 cells. Both compounds promoted Nrf2 nuclear translocation and induced HO-1 through p38- and JNK-dependent signaling, while protecting cells against tert-butyl hydroperoxide-induced oxidative injury [68]. Pharmacological manipulation of HO-1 supported a functional contribution of this enzyme, demonstrating that luteolin 7-O-glucoside can retain biological activity without prior conversion to the aglycone. In a complementary study, luteolin induced HO-1 through ERK1/2- and calcium-dependent signaling and reduced HMGB1, iNOS, NO, COX-2, and NF-κB activity in LPS-stimulated RAW 264.7 macrophages. Inhibition of HO-1 weakened these responses, providing evidence that HO-1 contributed directly to the anti-inflammatory effect [69].

More direct evidence of NF-κB/Nrf2 crosstalk was obtained in THP-1-derived macrophages undergoing pyroptosis. Luteolin reduced ROS accumulation, NLRP3 inflammasome activation, caspase-1 processing, gasdermin D cleavage, and IL-1β production. These changes were accompanied by increased Nrf2 nuclear translocation and HO-1 expression and reduced NF-κB p65 phosphorylation and nuclear localization. Partial reversal of the protective effect by an Nrf2 inhibitor supported a causal contribution of Nrf2 to the regulation of ROS-dependent pyroptosis [70].

Luteolin 7-O-glucuronide, which is structurally related to but chemically distinct from luteolin 7-O-glucoside, also reduced NO and iNOS expression in LPS-stimulated RAW 264.7 cells. The compound inhibited TAK1 and downstream NF-κB and MAPK signaling while promoting Nrf2-dependent antioxidant responses [71]. This evidence demonstrates that luteolin and selected conjugated derivatives can influence NF-κB, iNOS, ROS, Nrf2, and HO-1. However, glycosylation, cellular model, inflammatory stimulus, and concentration modify their activity.

### 6.3. Apigenin and Its Derivatives

Apigenin directly suppresses inflammatory mediator production in macrophage and monocyte models. In mouse macrophages stimulated with LPS and interferon gamma, apigenin reduced NO and PGE2 production by decreasing iNOS and COX-2 expression and increased peroxisome proliferator-activated receptor gamma (PPARγ) transcriptional activity [72]. In LPS-stimulated human monocytes and mouse macrophages, apigenin also reduced IL-1β, IL-8, and TNF production by suppressing NF-κB p65 phosphorylation, providing a more direct connection between apigenin and inflammatory transcriptional control [13].

In vivo evidence supports immune-regulatory activity but differs in its mechanistic proximity to macrophages. Dietary apigenin reduced NF-κB activity and inflammatory-cell infiltration in a tissue-dependent manner and improved metabolic alterations associated with systemic inflammatory activation [73]. A more direct link with macrophage phenotype was demonstrated in obesity-related inflammation. Apigenin bound to and activated PPARγ, inhibited p65 nuclear translocation, and favored a shift from an inflammatory M1-like state toward an M2-like profile. In high-fat-diet and genetically obese mice, these effects were accompanied by reduced macrophage infiltration and inflammatory cytokine production in adipose and hepatic tissues [74].

Conjugated apigenin derivatives also display macrophage activity, although their mechanisms are not interchangeable. Apigenin 7-O-glucoside, a form identified in *Buddleja globosa*, reduced hydrogen peroxide-induced ROS production and inhibited LPS-induced NF-κB/NLRP3/caspase-1 signaling in RAW 264.7 cells [75]. Apigenin 7-O-glucuronide reduced nitrite accumulation and TNF-α release at concentrations between 1 and 10 μg/mL, but iNOS, NF-κB, Nrf2, and downstream antioxidant targets were not evaluated [76]. Apigenin 7-glycoside additionally reduced iNOS, COX-2, TNF-α, IL-1β, and IL-6 in RAW 264.7 cells and attenuated LPS-induced acute lung injury in mice. These effects were associated with reduced MAPK phosphorylation, preservation of IκB, lower p65 activation, and increased HO-1 expression. Because Nrf2 activation was not measured directly, HO-1 induction alone cannot establish an Nrf2-dependent mechanism [77].

The available evidence indicates that apigenin and its conjugated derivatives regulate NF-κB, iNOS, COX-2, inflammatory cytokines, inflammasome signaling, ROS, and PPARγ-dependent macrophage responses. However, glycosylation and glucuronidation modify polarity, cellular uptake, metabolism, and biological availability, and the reported compounds differ in potency and molecular targets.

### 6.4. Quercetin and Quercetin Derivatives

Quercetin regulates early and downstream components of LPS-induced macrophage signaling. In RAW 264.7 cells, it inhibited Src and Syk activation and disrupted the association of the phosphatidylinositol 3-kinase p85 subunit with the TLR4/MyD88 complex. This interference reduced IRAK1, TRAF6, and TAK1 signaling; limited MAPK and AP-1 activation; suppressed the IKK/IκB-α/NF-κB cascade; and decreased the expression of iNOS, COX-2, and inflammatory mediators [78]. These findings indicate that quercetin acts at several interconnected signaling levels rather than functioning exclusively as a chemical antioxidant.

Quercetin also modifies macrophage redox regulation through HO-1. In RAW 264.7 cells it reduced NADPH oxidase-derived superoxide production without directly inhibiting the enzymatic complex. Pharmacological interference with HO-1 weakened this response, supporting a functional role for HO-1 in the regulation of oxidative activity [79]. However, Nrf2 activation was not evaluated, and HO-1 induction alone cannot establish an Nrf2-dependent mechanism.

Conjugated quercetin derivatives display related but non-equivalent biological activity. Quercetin and quercitrin, its 3-O-rhamnoside derivative, reduced intracellular ROS, NO production, and inflammatory mediator expression in LPS-stimulated RAW 264.7 macrophages at non-cytotoxic concentrations, while higher concentrations impaired cellular viability [80]. Quercetin-3-O-β-D-glucuronide also decreased NO and PGE2 production and reduced iNOS and COX-2 expression through inhibition of JNK and ERK phosphorylation. Nevertheless, it had limited effects on TNF-α and IL-1β and was generally less potent than the aglycone. Quercetin produced stronger activity but reduced viability at the highest concentration tested [81]. Quercitrin and quercetin glucuronide are chemically distinct from quercetin 3-O-glucoside, the derivative documented in *Buddleja globosa*, emphasizing that findings obtained with one conjugate cannot be transferred automatically to another.

Quercetin can additionally influence macrophage functional phenotype. Concentrations between 1 and 10 μM reduced LPS-induced iNOS, COX-2, IL-1β, TNF-α, IL-6, CCL2, and CXCL10 expression in RAW 264.7 cells, increased IL-10, and favored M2-associated responses [82]. Complementary experiments in microglial cells showed reduced ROS and increased HO-1, NQO1, and glutamate-cysteine ligase expression, although Nrf2 activation was not measured directly. The available evidence supports regulation of TLR4-associated signaling, NF-κB, iNOS, inflammatory cytokines, ROS, HO-1, and macrophage phenotype by purified quercetin.

### 6.5. Iridoids, Terpenoids, and Sterols

Catalposide provides constituent-level evidence relevant to the iridoid fraction of *Buddleja globosa*. In LPS-stimulated RAW 264.7 macrophages, purified catalposide reduced TNF-α, IL-1β, and IL-6 production; inhibited NF-κB activation and p65 nuclear translocation; and decreased LPS binding to the CD14 receptor [83]. These findings suggest regulation at an early stage of macrophage activation. However, the compound was isolated from *Catalpa ovata*, and its activity cannot be extrapolated automatically to other catalpol-related iridoids or to complete *Buddleja globosa* extracts.

Sterols identified in lipophilic botanical fractions also influence macrophage inflammatory and redox responses. In RAW 264.7 cells exposed to phorbol ester-induced oxidative stress, β-sitosterol improved the glutathione-to-oxidized-glutathione ratio and modified antioxidant enzyme activity, although Nrf2 was not evaluated [84]. Comparative testing of β-sitosterol, stigmasterol, campesterol, ergosterol, and ergosterol acetate showed compound-dependent reductions in phagocytosis, NO production, TNF-α release, iNOS, and COX-2, associated principally with reduced ERK phosphorylation [85]. β-Sitosterol-β-D-glucoside also reduced NO, TNF-α, IL-1β, and IL-6 in LPS-stimulated RAW 264.7 cells, but effects on NF-κB and Nrf2 were not determined [86]. These results demonstrate that structurally related sterols should not be considered mechanistically interchangeable.

Recent comparative evidence further showed that β-amyrin and stigmasterol reduced LPS-induced NO production and suppressed IL-1β and TNF-α release in RAW 264.7 and THP-1 cells within compound-specific non-cytotoxic concentration ranges [87]. Effects on IL-6 were limited, indicating that these compounds do not uniformly suppress all inflammatory mediators. Iridoids, triterpenoids, and sterols therefore provide complementary evidence of interference with LPS recognition, NF-κB and ERK signaling, iNOS activity, inflammatory cytokines, and intracellular redox balance. Nevertheless, most findings derive from purified compounds obtained from other botanical sources, and their concentrations and biological contribution within standardized *Buddleja globosa* preparations remain unknown.

## 7. Comparative Evidence from Related Botanical Extracts

Chemically characterized botanical extracts with related phenylethanoid and flavonoid profiles provide comparative models for evaluating macrophage immune-redox mechanisms. Their findings offer mechanistic guidance but cannot substitute for direct testing of standardized *Buddleja globosa* preparations.

### 7.1. Phenylethanoid- and Caffeic Acid Derivative-Rich Preparations

Chemically characterized *Forsythia suspensa* preparations provide a strong comparative model of coordinated inflammatory and cytoprotective regulation in macrophages. An HPLC-fingerprinted fruit decoction increased Nrf2 nuclear accumulation and induced HO-1, NQO1, and glutamate-cysteine ligase catalytic subunit expression in RAW 264.7 cells. The preparation also increased the ubiquitin-regulating protein A20 and suppressed NF-κB activation induced by LPS or TNF-α, demonstrating that a complete aqueous extract can simultaneously reinforce cytoprotective responses and restrict inflammatory signaling [88].

Stronger causal evidence was obtained with a polyphenol-rich *F. suspensa* extract in J774A.1 macrophages exposed to LPS and adenosine triphosphate. The extract reduced intracellular ROS, lactate dehydrogenase release, NLRP3 expression, caspase-1 activation, gasdermin D cleavage, and IL-1β production, while promoting Nrf2 nuclear accumulation and increasing HO-1 and NQO1 expression. Pharmacological inhibition of Nrf2 with ML385 weakened both the antioxidant response and the suppression of pyroptosis, supporting a functional role for Nrf2 rather than a simple parallel change in pathway markers [89]. These studies provide comparative evidence that chemically complex botanical preparations can coordinate Nrf2-dependent defenses and inflammatory signaling in macrophages.

### 7.2. Flavonoid- and Phenolic-Rich Preparations

Chemically characterized flavonoid-rich preparations provide comparative evidence that complete botanical mixtures can regulate both inflammatory and cytoprotective responses in macrophages. An ethyl acetate fraction from *Cistus* × *incanus* leaves, enriched in myricetin and quercetin derivatives, reduced IL-6 and COX-2 expression and decreased PGE2 production in LPS-stimulated RAW 264.7 macrophages at non-cytotoxic concentrations. The fraction also increased IL-10 expression, promoted Nrf2 nuclear translocation, and induced HO-1 [90]. These parallel changes are consistent with immune-redox modulation, although the absence of Nrf2 inhibition or genetic validation prevents confirmation that suppression of inflammatory mediators depended on Nrf2 activation.

A flavonoid-rich *Apios americana* leaf extract contained vicenin-2, schaftoside, vitexin, isoquercetin, and related apigenin and luteolin derivatives. In LPS-stimulated RAW 264.7 macrophages, the preparation reduced nitric oxide and inflammatory cytokine production and inhibited iNOS, COX-2, NF-κB, mitogen-activated protein kinase, and Akt–mTOR signaling. It also modified the Keap1–Nrf2 axis and pathways associated with autophagy and mitochondrial function [91]. The breadth of this response supports multitarget activity of the complete extract but prevents attribution of the effect to a single flavonoid.

A polyphenol-rich extract from *Chaenomeles japonica* leaves was evaluated at 10–50 μg/mL after confirming cellular viability. The preparation, characterized principally by chlorogenic acid, naringenin hexoside, and quercetin-related compounds, reduced *Nfkb1*, *Ptgs2*, and *Il1b* transcription in LPS-stimulated RAW 264.7 macrophages. It also decreased iNOS, COX-2, nitric oxide, TNF-α, IL-1β, and IL-6; preserved IκB-α; and reduced total and phosphorylated NF-κB p65 [92]. Although Nrf2 was not evaluated, the study provides a comparatively complete analysis of the NF-κB/iNOS axis under non-cytotoxic conditions. These models further support multitarget regulation of macrophage inflammatory and redox pathways by chemically complex flavonoid-rich preparations.

### 7.3. Extract–Constituent Comparisons and Interaction Effects

Direct comparison of complete extracts, enriched fractions, and purified constituents is necessary to determine whether a phytochemical marker reproduces the biological activity of the botanical preparation. Such comparisons should account for differences between extract mass concentrations and the molar concentrations of isolated compounds, as well as constituent enrichment, unequal cellular exposure, and interactions among metabolites.

An ethanolic *Echinacea* extract containing caffeic acid conjugates and alkylamides was compared with an alkylamide-enriched fraction, cichoric acid, and individual alkylamides. The complete extract and selected fractions reduced LPS-induced NF-κB activity and TNF-α production, whereas one isolated alkylamide increased TNF-α. Effects on nitric oxide were comparatively limited [60]. This opposing activity among constituents from the same preparation demonstrates that the response of a botanical extract cannot be predicted from the activity of a single marker compound.

A methanolic extract of *Cirsium maackii*, its ethyl acetate fraction, and luteolin 5-*O*-glucoside were evaluated in RAW 264.7 macrophages. The extract, active fraction, and isolated glycoside reduced LPS-induced nitric oxide production and iNOS and COX-2 expression. They also limited tert-butyl hydroperoxide-induced ROS generation at non-cytotoxic concentrations [93]. Although the isolated glycoside reproduced part of the extract response, the absence of NF-κB and Nrf2 measurements prevented determination of whether the preparations acted through the same upstream mechanism.

A methanolic extract of *Eriosema montanum* was similarly compared with chromatographic fractions and isolated genistin. The complete extract, several fractions, and genistin inhibited nitric oxide production without significant cytotoxicity at the initially tested concentrations. However, the most active fraction produced substantially greater nitric oxide and 15-lipoxygenase inhibition than either the complete extract or purified genistin. The extract also reduced intracellular ROS more effectively than genistin, demonstrating that the isolated constituent did not reproduce the complete redox response of the botanical preparation [94].

These comparisons show that an abundant or chemically convenient marker may reproduce only part of an extract response, display lower potency than an enriched fraction, or even produce an effect in the opposite direction. Mechanistic studies of *Buddleja globosa* should therefore evaluate the standardized extract alongside verbascoside, luteolin and apigenin glycosides, quercetin derivatives, and selected lipophilic constituents at concentrations corresponding to their quantified abundance in the extract. Reconstituted mixtures and preparations selectively depleted of major markers would provide stronger evidence of additive, antagonistic, or interaction-dependent effects than testing each constituent independently.

## 8. Complementary Biological Activities and Pharmaceutical Translation

The therapeutic relevance of *Buddleja globosa* also depends on whether its biological activity can be translated into tissue-repair and delivery systems. This section examines wound-healing evidence, formulation strategies, and the extent to which observed outcomes can be attributed to the extract rather than to the vehicle or biomaterial.

### 8.1. Wound Healing and Tissue Regeneration

Direct wound-healing evidence was initially obtained with a standardized hydroalcoholic *Buddleja globosa* leaf extract in Sprague-Dawley rats. Oral administration for up to 12 days did not produce detectable hematological or clinical chemistry alterations, while topical treatment accelerated the macroscopic progression of wound repair and modified COX-2 expression, cellular proliferation, epidermal thickness, and tissue morphology [95]. The response was associated with a shorter inflammatory phase, but macrophage infiltration, cytokine production, NF-κB activity, iNOS expression, and Nrf2 signaling were not directly examined.

More recent studies incorporated standardized *Buddleja globosa* extracts into gelatin, chitosan-, and hyaluronic acid-based scaffolds. In a murine wound model infected with a dual-species biofilm of *Pseudomonas aeruginosa* and *Staphylococcus aureus*, an intermediate extract loading produced the most favorable combination of wound closure and complete re-epithelialization. The highest loading increased selected antimicrobial effects but reduced pore size, swelling capacity, and mechanical resistance and did not further improve wound repair [96]. A related extract-enriched biocomposite was evaluated in human neonatal dermal fibroblasts and full-thickness ischemic wounds in aged rats. The formulation supported fibroblast growth and increased VEGF and CXCL12 secretion. Although wound closure was not significantly accelerated, treated wounds displayed greater vascular density, reduced epithelial hyperplasia, and improved dermal tissue organization [97].

These studies indicate that *Buddleja globosa* may influence the progression and quality of tissue repair, but the observed response depends on extract loading, scaffold architecture, cytocompatibility, antimicrobial performance, and the characteristics of the wound model. The available evidence is compatible with inflammation and redox modulation but does not establish that the regenerative effects depend specifically on macrophage NF-κB/iNOS–Nrf2 regulation.

### 8.2. Antimicrobial and Antibiofilm Activity

The antimicrobial activity of complete *Buddleja globosa* preparations has been inconsistent and strongly dependent on extraction and formulation conditions. A comparative investigation of medicinal plants used by Huilliche communities found no relevant antimicrobial activity for a methanolic *Buddleja globosa* extract against the tested microorganisms, including several *Pseudomonas aeruginosa* strains [98]. In contrast, the standardized hydroalcoholic extract BG-126 and a spray-dried preparation derived from the same extract inhibited *P. aeruginosa* proliferation. The spray-dried preparation produced complete inhibition at a lower phenolic-equivalent concentration than BG-126, whereas the effects on biofilm formation differed from those observed against planktonic bacteria. Ethanol and the solubilizing excipients also modified biofilm development independently, demonstrating that antimicrobial outcomes cannot be attributed to the botanical preparation without matched formulation controls [52].

Constituent-level evidence supports the antimicrobial plausibility of phenylethanoid-rich preparations. Verbascoside isolated from the related species *Buddleja cordata* exerted bacteriostatic and bactericidal activity against *Staphylococcus aureus* and interfered predominantly with leucine incorporation and protein synthesis [99]. However, this mechanism cannot establish that verbascoside accounts for the activity of complete *Buddleja globosa* extracts or their delivery systems. Within the present review, antimicrobial effects are therefore relevant principally because microbial burden and biofilm formation can prolong wound inflammation, not because they demonstrate direct regulation of macrophage NF-κB/iNOS–Nrf2 signaling.

### 8.3. Formulation and Delivery Strategies

The formulation of *Buddleja globosa* determines the solubility, stability, and biological exposure of its constituents. The standardized BG-126 extract, originally maintained in a solution containing 53% ethanol, was converted into spray-dried powders with or without polyvinylpyrrolidone and Soluplus^®^ as solubility-enhancing excipients. The excipient-containing preparations reached higher extract-equivalent concentrations in water, whereas the spray-dried extract without excipients displayed greater antibacterial potency than the original hydroalcoholic preparation [52]. These findings demonstrate that processing and excipient selection can modify biological performance even when formulations originate from the same standardized extract.

BG-126 has also been incorporated into porous gelatin–chitosan–hyaluronic acid scaffolds. Raman mapping indicated a relatively homogeneous distribution of the extract, but fibroblast viability was strongly influenced by gelatin content, while chitosan contributed independently to antimicrobial activity [97]. Electrospun polycaprolactone fibers containing both *Buddleja globosa* and *Gunnera tinctoria* extracts showed favorable morphology, mechanical performance, and fibroblast cytocompatibility [100]. However, the use of two botanical extracts prevented attribution of the response specifically to *Buddleja globosa*. In infected-wound scaffolds containing increasing BG-126 concentrations, an intermediate extract loading produced a more favorable healing response than the highest loading, which altered pore size, swelling capacity, and mechanical resistance [96].

Current platforms range from water-dispersible powders to porous biopolymeric scaffolds and electrospun fibers. Nevertheless, release kinetics, constituent stability after processing, retention of phytochemical markers, carrier degradation, local tissue exposure, and macrophage compatibility remain insufficiently characterized. Future formulations should quantify the release of verbascoside and other representative constituents and determine whether the concentrations delivered to cells and tissues remain non-cytotoxic and preserve the proposed immune-redox activity.

### 8.4. Translational Limitations

Translation of *Buddleja globosa* into a reproducible therapeutic intervention requires definition of the complete active preparation rather than reliance on total phenolic content or a single marker compound. Botanical identity, plant organ, provenance, harvesting and postharvest conditions, extraction solvent, drug-to-extract ratio, manufacturing process, chromatographic fingerprint, quantitative marker ranges, contaminants, and stability should be controlled collectively. Regulatory guidance for botanical and herbal medicinal products similarly emphasizes characterization of the starting material, manufacturing process, complete herbal preparation, and batch consistency [101,102].

The biologically effective exposure produced by *Buddleja globosa* preparations remains unknown. Available studies generally report nominal extract concentration, total phenolics, catechin equivalents, or verbascoside content without determining the concentrations of individual constituents that reach cells or tissues. Pharmacokinetic analysis of purified acteoside in rats demonstrated rapid distribution and elimination and an absolute oral bioavailability of approximately 1% [103]. Moreover, glucuronide conjugates were the predominant luteolin-derived forms detected after administration of luteolin and several luteolin glycosides [104]. Macrophages in vivo may therefore encounter circulating conjugates or metabolites rather than the original aglycones and glycosides used in cell-culture experiments. These pharmacokinetic differences indicate that concentrations producing biological effects in vitro should not automatically be assumed to be physiologically achievable in vivo. Their translational relevance should instead be evaluated against plasma and target-tissue exposures to parent compounds, conjugates, and active metabolites rather than nominal in vitro concentrations alone [103,104].

Oral and topical applications should therefore be considered as distinct exposure scenarios. Following oral administration, systemic macrophages are likely to encounter absorbed metabolites and conjugated forms at concentrations that may differ substantially from those used in vitro. In contrast, topical wound formulations may generate higher local exposure to extract constituents, but the concentrations reaching macrophages and other resident cells will depend on formulation composition, release kinetics, carrier degradation, and tissue penetration. These routes should therefore not be interpreted as biologically equivalent exposure conditions.

Safety information is limited to short-term animal studies and viability assessments in fibroblasts, erythrocytes, Vero cells, and other experimental systems. These findings do not establish safety across routes, formulations, or prolonged exposure. Further development should evaluate genotoxicity, dermal irritation and sensitization, repeated-dose toxicity, local tissue accumulation, interactions with drug-metabolizing enzymes and transporters, and contaminants such as pesticides, microorganisms, mycotoxins, residual solvents, and elemental impurities [102].

No controlled human clinical study identified in this review evaluated the efficacy, pharmacokinetics, dose–response relationship, or treatment-related adverse events of a chemically standardized *Buddleja globosa* extract. Consequently, the available evidence remains preclinical and does not yet support conclusions regarding clinical efficacy, optimal dosing, systemic exposure, or long-term safety in humans. Translation will require a reproducible preparation and route-specific dosage form, followed by pharmacokinetic and toxicological assessment, confirmation of biological activity in relevant human cells, and clinical evaluation using compositionally comparable batches. For wound applications, outcomes should include microbial burden, inflammatory duration, vascularization, re-epithelialization, tissue architecture, scarring, and treatment tolerability rather than wound closure alone. To distinguish direct observations from constituent-based and comparative mechanistic inferences, the evidence linking *Buddleja globosa* to macrophage NF-κB/iNOS–Nrf2 regulation is summarized according to experimental proximity, pathway coverage, and principal interpretive limitations in Table 2.

## 9. Conclusions

Direct studies support antioxidant, inflammation-modulating, membrane-protective, and tissue-repair-associated activities of *Buddleja globosa* preparations. However, the available evidence does not demonstrate that a complete *Buddleja globosa* extract directly regulates the macrophage NF-κB/iNOS–Nrf2 network. Most mechanistic support derives from purified phenylethanoids and flavonoids, particularly verbascoside-related compounds and luteolin, apigenin, and quercetin derivatives, which influence TLR4-associated signaling, NF-κB, iNOS, inflammatory cytokines, reactive oxygen species, Nrf2, and HO-1 in macrophage or related models. These constituent-level effects cannot be transferred automatically to a chemically complex extract. The central research priority is therefore not to confirm that *Buddleja globosa* possesses general antioxidant or anti-inflammatory activity, but to determine whether a chemically standardized leaf extract produces coordinated immune-redox regulation in macrophages at non-cytotoxic concentrations. This requires direct comparison of the complete extract, defined fractions, and quantified constituents, together with pathway-specific evaluation of NF-κB, iNOS, Nrf2, and their downstream responses. Formulation studies demonstrate that *Buddleja globosa* can be incorporated into pharmaceutically relevant delivery systems, although extract loading, excipients, release behavior, and biomaterial properties strongly influence biological performance. Resolving these mechanistic and compositional uncertainties is necessary before *Buddleja globosa* can be considered a reproducible macrophage-targeted immune-redox intervention.

## Figures and Tables

**Table 1:** Preparation-dependent phytochemical characteristics of *Buddleja globosa* leaf extracts and their implications for macrophage immune-redox studies.

Preparation	Representative Constituents or Markers	Analytical Approach	Relevance to Macrophage Studies	Principal Limitation	References
Polar methanolic or hydroalcoholic leaf extracts	Verbascoside, luteolin 7-O-glucoside, apigenin 7-O-glucoside, quercetin derivatives, rutin, and caffeic acid	Chromatographic separation, HPLC, or UHPLC-MS-based profiling	Contain the phenylethanoid and flavonoid constituents most closely associated with the proposed modulation of NF-κB/iNOS and Nrf2 signaling	Composition varies with harvesting, solvent, and fractionation; constituent abundance does not establish biological contribution	[10,46,48,53]
Aqueous leaf extracts	Chemically unresolved or partially characterized mixtures of phenolic acids and flavonoids	Phytochemical screening, total phenolic content, and cellular antioxidant assays	Relevant to traditional preparations and concentration-dependent cellular antioxidant effects	Incomplete chemical characterization and the use of non-macrophage models limit mechanistic interpretation	[54]
Sequential nonpolar leaf fractions	Triterpenoids, sterols, and other lipophilic constituents	Solvent fractionation followed by chromatographic or spectroscopic characterization	Demonstrate that nonpolar fractions possess chemical and biological properties different from phenolic-rich extracts	Direct evidence concerning macrophage NF-κB/iNOS–Nrf2 signaling is unavailable	[10]
Standardized or formulated hydroalcoholic leaf extracts	Verbascoside and total phenolic content used as quantitative markers	HPLC, extraction-yield determination, spray drying, and physicochemical characterization	Allow assessment of how processing and solubility influence the extract concentration delivered to biological systems	Drying, residual solvent, and excipients may alter stability and biological performance independently of the extract	[52]

Abbreviations: HPLC, high-performance liquid chromatography; iNOS, inducible nitric oxide synthase; NF-κB, nuclear factor kappa B; Nrf2, nuclear factor erythroid 2-related factor 2; UHPLC–MS, ultra-high-performance liquid chromatography coupled to mass spectrometry.

**Table 2:** Evidence classification for macrophage NF-κB/iNOS–Nrf2 modulation by *Buddleja globosa* extracts, associated constituents, comparative botanical preparations, and translational systems.

Evidence Category	Preparation or Intervention	Experimental Model	NF-κB/iNOS-Related Findings	Nrf2 and Redox-Related Findings	Mechanistic Interpretation and Principal Limitation	References
I. Direct *B. globosa* evidence	Complete or fractionated leaf extracts	Cell-free assays; fibroblasts, erythrocytes, platelets, and rodent inflammation models	Anti-inflammatory activity, inhibition of eicosanoid-generating enzymes, and modification of intracellular signaling were demonstrated, but NF-κB, *NOS2*/iNOS, NO, and macrophage cytokines were not directly evaluated in a macrophage model	Radical scavenging and protection against oxidative injury to lipids, proteins, fibroblasts, and cellular membranes were reported; Nrf2 activation was not examined	Establishes direct antioxidant and inflammation-modulating activity of *B. globosa*, but provides no direct evidence that the complete extract regulates the macrophage NF-κB/iNOS–Nrf2 network	[14,47,54,55,57,58,59]
II. Constituent-based evidence	Verbascoside, acteoside, and isoverbascoside	THP-1 cells, RAW 264.7 cells, primary bone marrow macrophages, mouse macrophages, and C2C12 cells	Reduced TLR4 dimerization, NF-κB and MAPK signaling, iNOS, NO, caspase-1 activation, and inflammatory cytokine production	Reduced ROS and altered antioxidant enzyme activity; Nrf2 nuclear translocation and HO-1 induction were demonstrated primarily outside macrophage models	Provides strong mechanistic plausibility, particularly for upstream TLR4 regulation by isoverbascoside, but purified phenylethanoids do not reproduce the chemical complexity or constituent concentrations of the complete extract	[62,63,64,65,66]
II. Constituent-based evidence	Luteolin and luteolin conjugates	MH-S macrophages, RAW 264.7 cells, and THP-1-derived macrophages	Reduced NF-κB and AP-1 activity, iNOS, COX-2, NO, inflammatory cytokines, and NLRP3-dependent pyroptosis	Increased Nrf2 nuclear translocation and HO-1 expression; pharmacological inhibition provided evidence that Nrf2 or HO-1 contributed functionally to selected responses	Provides the most direct constituent-level evidence of NF-κB/Nrf2 crosstalk in macrophages; however, aglycones, glucosides, and glucuronides are chemically and biologically non-equivalent	[67,68,69,70,71]
II. Constituent-based evidence	Apigenin and apigenin conjugates	Human monocytes, mouse macrophages, RAW 264.7 cells, and inflammatory animal models	Reduced p65 phosphorylation and nuclear localization, iNOS, COX-2, NO, cytokines, and NF-κB/NLRP3/caspase-1 signaling; PPARγ-dependent changes in macrophage phenotype were also reported	Reduced ROS and increased HO-1 in selected studies, but direct Nrf2 activation or pathway dependency was not consistently evaluated	Supports broad inflammatory regulation, but evidence differs among apigenin, glucosides, and glucuronides, and HO-1 induction alone does not establish an Nrf2-dependent mechanism	[13,72,73,74,75,76,77]
II. Constituent-based evidence	Quercetin and quercetin derivatives	RAW 264.7 macrophages and related myeloid models	Interfered with TLR4/MyD88-associated signaling, IKK/IκB-α/NF-κB activation, iNOS, COX-2, NO, and inflammatory cytokine expression	Reduced NADPH oxidase-derived ROS and increased HO-1, NQO1, and related antioxidant responses; direct Nrf2 validation remained limited	Demonstrates multitarget regulation, but quercetin, quercitrin, glucuronides, and the quercetin 3-O-glucoside documented in *B. globosa* cannot be treated as interchangeable	[78,79,80,81,82]
II. Complementary constituent evidence	Catalposide, β-sitosterol, phytosterols, β-amyrin, and stigmasterol	RAW 264.7 and THP-1 cells	Reduced LPS binding, NF-κB activation, ERK signaling, iNOS, NO, TNF-α, IL-1β, and other inflammatory mediators	Modified glutathione balance and antioxidant enzyme activity; direct Nrf2 activation was not established	Suggests that iridoids, triterpenoids, and sterols may contribute to extract activity, but most compounds were isolated from other botanical sources and their abundance in *B. globosa* preparations remains uncertain	[83,84,85,86,87]
III. Comparative botanical evidence	Phenylethanoid-, flavonoid-, and polyphenol-rich extracts from *Forsythia suspensa*, *Cistus* × *incanus*, *Apios americana*, and *Chaenomeles japonica*	RAW 264.7 and J774A.1 macrophages	Reduced NF-κB, iNOS, COX-2, NO, inflammatory cytokines, NLRP3 activation, and pyroptosis-related markers	Increased Nrf2 nuclear accumulation, HO-1, and NQO1; Nrf2 inhibition with ML385 supported pathway dependency in the F. suspensa model	Demonstrates that chemically complex extracts can coordinately regulate inflammatory and cytoprotective pathways in macrophages, but botanical and compositional differences prevent extrapolation to *B. globosa*	[88,89,90,91,92]
III. Extract–constituent comparisons	Complete extracts, enriched fractions, and purified constituents from *Echinacea*, *Cirsium maackii*, and *Eriosema montanum*	RAW 264.7 macrophages	Extracts, fractions, and isolated markers produced different effects on NF-κB, iNOS, NO, and inflammatory mediator production; some isolated constituents produced weaker or opposing responses	Complete extracts or active fractions sometimes reduced intracellular ROS more effectively than isolated marker compounds	Provides direct evidence that a phytochemical marker may reproduce only part of an extract response and supports matched extract–fraction–constituent comparisons for *B. globosa*	[60,93,94]
IV. Translational evidence	Spray-dried extracts, gelatin–chitosan–hyaluronic acid scaffolds, and electrospun botanical fibers	Human dermal fibroblasts, bacterial biofilms, and infected or ischemic wound models	Improved selected antimicrobial, angiogenic, re-epithelialization, and tissue-organization outcomes; macrophage infiltration and NF-κB/iNOS signaling were not evaluated	Nrf2 activation, intracellular redox responses, and extract-specific immune-redox activity were not measured	Demonstrates formulation feasibility, but biological performance depends on extract loading, excipients, polymer composition, release behavior, and the delivery matrix; effects cannot be attributed exclusively to the extract	[52,96,97,100]

Abbreviations: AP-1, activator protein 1; COX-2, cyclooxygenase-2; HO-1, heme oxygenase-1; IKK, inhibitor of nuclear factor kappa B kinase; iNOS, inducible nitric oxide synthase; LPS, lipopolysaccharide; MAPK, mitogen-activated protein kinase; MyD88, myeloid differentiation primary response 88; NF-κB, nuclear factor kappa B; NLRP3, nucleotide-binding oligomerization domain-like receptor family pyrin domain-containing 3; NO, nitric oxide; NOS2, nitric oxide synthase 2; NQO1, NAD(P)H quinone oxidoreductase 1; Nrf2, nuclear factor erythroid 2-related factor 2; PPARγ, peroxisome proliferator-activated receptor gamma; ROS, reactive oxygen species; TLR4, toll-like receptor 4; TNF-α, tumor necrosis factor alpha.

## Data Availability

No new data were created or analyzed in this study. Data sharing is not applicable to this article.

## Abbreviations

The following abbreviations are used in this manuscript:

Akt	protein kinase B
AP-1	activator protein 1
ARE	antioxidant response element
BG-126	standardized hydroalcoholic *Buddleja globosa* extract
C2C12	murine myoblast cell line
CCL2	C-C motif chemokine ligand 2
CD14	cluster of differentiation 14
COX-2	cyclooxygenase-2
CXCL10	C-X-C motif chemokine ligand 10
CXCL12	C-X-C motif chemokine ligand 12
DPPH	2,2-diphenyl-1-picrylhydrazyl
ERK1/2	extracellular signal-regulated kinases 1 and 2
GCLC	glutamate-cysteine ligase catalytic subunit
HMGB1	high-mobility group box 1
*HMOX1*	heme oxygenase 1 gene
HO-1	heme oxygenase-1
HPLC	high-performance liquid chromatography
IFN-γ	interferon gamma
IKK	inhibitor of nuclear factor kappa B kinase
IκB-α	inhibitor of nuclear factor kappa B alpha
IL-1β	interleukin 1 beta
IL-4	interleukin 4
IL-6	interleukin 6
IL-8	interleukin 8
IL-10	interleukin 10
IL-13	interleukin 13
iNOS	inducible nitric oxide synthase
IRAK1	interleukin-1 receptor-associated kinase 1
IRF3	interferon regulatory factor 3
J774A.1	murine macrophage-like cell line
JNK	c-Jun N-terminal kinase
Keap1	Kelch-like ECH-associated protein 1
LPS	lipopolysaccharide
MAPK	mitogen-activated protein kinase
MD-2	myeloid differentiation factor 2
MH-S	murine alveolar macrophage cell line
ML385	selective Nrf2 inhibitor
mTOR	mechanistic target of rapamycin
MyD88	myeloid differentiation primary response 88
NADPH	nicotinamide adenine dinucleotide phosphate
NF-κB	nuclear factor kappa B
*NFE2L2*	nuclear factor erythroid 2-like 2 gene
NLRP3	nucleotide-binding oligomerization domain-like receptor family pyrin domain-containing 3
NO	nitric oxide
*NOS2*	nitric oxide synthase 2 gene
NQO1	NAD(P)H quinone oxidoreductase 1
Nrf2	nuclear factor erythroid 2-related factor 2
ONOO^−^	peroxynitrite
p65	RelA subunit of NF-κB
PGE2	prostaglandin E2
PPARγ	peroxisome proliferator-activated receptor gamma
RAW 264.7	murine macrophage-like cell line
RNS	reactive nitrogen species
ROS	reactive oxygen species
Src	Src family tyrosine kinase
Syk	spleen tyrosine kinase
TAK1	transforming growth factor beta-activated kinase 1
THP-1	human monocytic cell line
TLR4	toll-like receptor 4
TNF-α	tumor necrosis factor alpha
TRAF6	tumor necrosis factor receptor-associated factor 6
TRIF	TIR-domain-containing adapter-inducing interferon-β
UHPLC-MS	ultra-high-performance liquid chromatography coupled to mass spectrometry
VEGF	vascular endothelial growth factor
